# Mitochondrial DNA haplogroup T is associated with coronary artery disease and diabetic retinopathy: a case control study

**DOI:** 10.1186/1471-2350-10-35

**Published:** 2009-04-21

**Authors:** Barbara Kofler, Edith E Mueller, Waltraud Eder, Olaf Stanger, Richard Maier, Martin Weger, Anton Haas, Robert Winker, Otto Schmut, Bernhard Paulweber, Bernhard Iglseder, Wilfried Renner, Martina Wiesbauer, Irene Aigner, Danijela Santic, Franz A Zimmermann, Johannes A Mayr, Wolfgang Sperl

**Affiliations:** 1 Department of Pediatrics, University Hospital Salzburg, Paracelsus Medical University, Salzburg, Austria; 2 Department of Cardiac Surgery, University Hospital Salzburg, Paracelsus Medical University, Salzburg, Austria; 3 Department of Ophthalmology, Medical University Graz, Graz, Austria; 4 Department of Occupational Medicine, Medical University of Vienna, Vienna, Austria; 5 Department of Internal Medicine, University Hospital Salzburg, Paracelsus Medical University, Salzburg, Austria; 6 Department of Geriatrics, University Hospital Salzburg, Paracelsus Medical University, Salzburg, Austria; 7 Clinical Institute of Medical and Chemical Laboratory Diagnostics, Medical University Graz, Graz, Austria

## Abstract

**Background:**

There is strong and consistent evidence that oxidative stress is crucially involved in the development of atherosclerotic vascular disease. Overproduction of reactive oxygen species (ROS) in mitochondria is an unifying mechanism that underlies micro- and macrovascular atherosclerotic disease. Given the central role of mitochondria in energy and ROS production, mitochondrial DNA (mtDNA) is an obvious candidate for genetic susceptibility studies on atherosclerotic processes. We therefore examined the association between mtDNA haplogroups and coronary artery disease (CAD) as well as diabetic retinopathy.

**Methods:**

This study of Middle European Caucasians included patients with angiographically documented CAD (n = 487), subjects with type 2 diabetes mellitus with (n = 149) or without (n = 78) diabetic retinopathy and control subjects without clinical manifestations of atherosclerotic disease (n = 1527). MtDNA haplotyping was performed using multiplex PCR and subsequent multiplex primer extension analysis for determination of the major European haplogroups. Haplogroup frequencies of patients were compared to those of control subjects without clinical manifestations of atherosclerotic disease.

**Results:**

Haplogroup T was significantly more prevalent among patients with CAD than among control subjects (14.8% vs 8.3%; p = 0.002). In patients with type 2 diabetes, the presence of diabetic retinopathy was also significantly associated with a higher prevalence of haplogroup T (12.1% vs 5.1%; p = 0.046).

**Conclusion:**

Our data indicate that the mtDNA haplogroup T is associated with CAD and diabetic retinopathy in Middle European Caucasian populations.

## Background

Mitochondria are the primary source of endogenous reactive oxygen species (ROS), generated through oxidative phosphorylation (OXPHOS) as a by-product of ATP synthesis. It is a generally accepted principle that in normal cells, ROS form a regular part of diverse redox signalling pathways, whereas in many types of chronic and cardiovascular diseases, ROS production exceeds antioxidant capacity with damaging effects on cell function and structure [1]. Therefore, mitochondrial dysfunction has been implicated in the pathogenesis of several diseases characterised by free radical-induced damage. Given the central role of mitochondria in energy and ROS production, mitochondrial DNA (mtDNA) is an obvious candidate for genetic susceptibility studies on atherosclerotic processes. The extranuclear mitochondrial genome is highly polymorphic and this variation could contribute to the range of complex traits in energy metabolism. Four of the enzymatic complexes that constitute the oxidative phosphorylation system (OXPHOS) are partially encoded by the mtDNA. Rare pathogenic mutations of the mtDNA, that either impair mitochondrial protein synthesis or impair proteins encoded by the mtDNA have been associated with more than 70 human diseases [2].

During evolution, a number of mutations have accumulated in the mtDNA [3]. Mutations acquired during evolution have subdivided the human population into a number of discrete, region specific, mitochondrial clades or haplogroups. MtDNA haplogroups are defined on the basis of specific single nucleotide polymorphisms (SNPs) scattered throughout the mitochondrial genome. In populations of European ancestry, nine such haplogroups with frequencies of at least 1% have been described [4]. Emerging evidence suggests that different mtDNA haplogroups are associated with subtle differences in OXPHOS capacity and the generation of ROS [5,6]. Recent evidence suggests that mtDNA haplogroups have functional consequences, being linked to longevity [7], sperm motility [5], certain types of cancer [8] and affecting the risk of individuals developing specific late-onset neurodegenerative diseases [9].

Diabetic retinopathy is caused by alterations in the retinal microvasculature leading to breakdown of the blood-retina barrier and pathological angiogenesis [10] and is one of the primary causes of visual loss worldwide [11]. Diabetic retinopathy and CAD are two vascular complications of type 2 diabetes mellitus, representing examples of microangiopathy and macroangiopathy, respectively. Retinopathy is also an early and frequent sign of other vascular complications and is strongly associated with the development of CAD [12,13]. This indicates that CAD and diabetic retinopathy share common risk factors and pathological mechanisms, such as diabetes and inflammation, which both exhibit increased ROS production [14,15].

It has been shown that oxidative stress due to disturbance in the balance between production of ROS and antioxidant defence plays a vital role in the pathogenesis of coronary atherosclerosis and its complications [15].

We aimed to investigate the association of mtDNA haplogroups with CAD as well as diabetic retinopathy.

## Methods

### Patients and control subjects

Data from 2352 Caucasian subjects enrolled in three surveys in central and southern Austria were analysed (Table 1). The study was conducted according to the Austrian Gene Technology Act and complied with the Declaration of Helsinki. All subjects gave written informed consent before entering the study.

**Table 1:** Characteristics of the study populations.

	Controls	Patients with heart disease		Patients with diabetes	
		CAD	Valvular Heart Disease	No Retinopathy	Retinopathy
	n = 1527	n = 487	n = 111	n = 78	n = 149

Mean (SD*) age (years)	51.5 (6.1)	63.4 (11.3)	64.9 (12.9)	76.9 (9.8)	71.1 (11.2)
Male (%)	64.7	76.4	54.1	47.4	36.9
Mean (SD*) BMI^† ^(kg/m^2^)	26.6 (4.1)	27.3 (3.7)	26.3 (4.5)	27.5 (4.5)	27.7 (5.3)
History of myocardial infarction (%)	0	50.5	0	7.7	14.8
Diagnosis of diabetes (%)	0	25.5	16.2	100	100
Hypertension (%)	13.7	67.6	59.5	75.6	71.8
Nonsmoker (%)	65.3	48.7	72.4	n.a.^‡^	n.a.^‡^
Former smoker (%)	13.5	37.5	14.3	n.a.^‡^	n.a.^‡^
Current smoker (%)	21.2	13.8	13.3	n.a.^‡^	n.a.^‡^

*SD: standard deviation; ^†^BMI: body mass index;^‡^n.a. = not available.

Patients with heart diseases (n = 598) were recruited in Salzburg and Graz. Four hundred and eighty-seven individuals had angiographically documented CAD with at least one of the main coronary arteries showing ≥ 50% stenosis. CAD was angiographically excluded in 111 patients with valvular heart disease.

The study population of patients with diabetes (n = 227) was recruited at the University Hospital of Graz and consisted of patients with (n = 149) and without diabetic retinopathy (n = 78). Presence and severity of diabetic retinopathy were assessed by fundus examination. Diabetic retinopathy was graded according to the modified "Early Treatment Diabetic Retinopathy Study" (ETDRS) retinopathy scale as no apparent retinopathy, non-proliferative, and proliferative diabetic retinopathy.

The control population consisted of 1527 unrelated individuals as previously described in detail [16]. Briefly, the cohort comprised 988 men between 39 and 66 years of age and 539 women between 39 and 67 years of age who were recruited in the Salzburg Atherosclerosis Prevention Program (SAPHIR) [16]. Exclusion criteria for participation in this study were a history of CAD, heart failure, cerebrovascular disease, peripheral vascular disease, haemodynamically relevant heart valve disease, chronic disease (of liver or kidney, autoimmune disorders, malignant cancer, haematologic disorders, endocrinopathies, diabetes mellitus) and morbid obesity. Laboratory methods have been described previously [16]. The haplogroup frequency in 1172 individuals of the control cohort was reported [17]. The study was approved by the Ethics Committee of the Medical Association of Salzburg.

### MtDNA analysis

Venous blood was collected in 5 ml EDTA tubes; total DNA was isolated with a Nucleospin Blood Kit (Macherey-Nagel) and stored at 4°C. A hierarchical system for mtDNA haplogrouping that combines multiplex PCR amplifications, multiplex single-base primer extensions, and capillary-based electrophoretic separation for analysing ten haplogroup-diagnostic mitochondrial SNPs (mtSNPs) was used to determine the haplogroup distribution of the most common European haplogroups H, U, J, T, K, I, V, W and X, as described previously [17]. Haplogroups that could not be assigned to one of the nine major European haplogroups by the SNP combination were designated as "others". From the 2387 subjects investigated, genotyping failed in 0.21% of the samples (n = 5). PCR amplification failed due to degradation of DNA in 1.26% of samples (n = 30).

### Statistical analysis

Frequencies of all mitochondrial haplogroups in CAD patients and in controls were tested for independency using Pearson chi-square statistics and Fisher's exact test as appropriate. Similarly, frequencies of mitochondrial haplogroups were compared between patients with heart disease other than CAD and controls. For further analysis only low frequency haplogroups (K, W, V, I, X) were excluded from further analyses. Age and gender adjusted odds ratios and 95% confidence intervals were then calculated to express the strength of the associations between the haplogroups and phenotypes. The following variables were also considered as potential confounders and tested in logistic regression models: hypertension (yes/no), body mass index (BMI), and smoking (never, former smoker, current smoker). Covariates that had a significance level of less than 0.1 were retained to create the best-fitting model based on Akaike's information criterion. If one category of an independent variable had a significance of 0.1 or less, all parts of the categorical variable were retained in the best-fitting model. P-values were corrected for multiple comparison by Bonferroni analysis (required significance level = 0.05/number of comparisons), leading to a new required significance level of <0.008 [number of comparisons = 6 (3 × 2) for 3 haplogroups compared to haplogroup H and to two diseases (CAD and Valvular Heart Disease)].

The same methods as described above were applied to calculate associations between mitochondrial haplogroups and the presence of retinopathy among patients with diabetes. Sex-adjusted odds ratios and 95% confidence intervals were then tested for the potential confounding effect of age at onset of diabetes (years), duration of diabetes (months), levels of HbA1c (%) measured at the time of recruitment, medication (insulin, oral antidiabetics), history of myocardial infarction (yes/no), and hypertension (yes/no). The best-fitting model was created in the same manner as for the heart disease population. P-values were corrected for multiple comparison by Bonferroni analysis (required significance level = 0.05/number of comparisons), leading to a new required significance level of <0.017 (number of comparisons = 3 for 3 haplogroups compared to haplogroup H).

All analyses were performed using SPSS 15.0 student version (SPSS GmbH Software, 80339 Munich, Germany) and Stata/SE 10.0 (Stata Corporation, College Station, TX, USA).

## Results

Clinical characteristics of patients and controls are shown in Table 1. All nine European haplogroups were observed in our sample, as expected (Table 2). Frequencies in the control group did not differ significantly from those previously reported for neighbouring European Caucasian populations [17,18].

**Table 2:** Frequencies (%) of mitochondrial haplogroups in cases and controls.

Haplogroup	Controls	Patients with heart diseases		Patients with diabetes	
		CAD	Valvular Heart Disease	No Retinopathy	Retinopathy
	n = 1527	n = 487	n = 111	n = 78	n = 149

H	43.6	37.8	46.0	52.5	43.6
U	15.5	15.0	16.2	11.5	14.8
J	11.4	10.9	12.6	5.1	10.7
T	8.3	14.8	9.0	5.1	12.1
K	5.2	3.1	0.9	3.9	3.4
W	2.1	1.8	1.8	0.0	2.0
V	1.8	3.1	0.9	2.6	2.0
I	1.0	1.2	1.8	1.3	0.7
X	1.3	3.1	1.8	3.9	2.0
others	9.8	9.2	9.0	14.1	8.7

Frequencies of mitochondrial haplogroups in patients with CAD differed significantly from those in healthy controls. The frequency of haplogroup T in controls was 8.3% and in patients with CAD 14.8%. After adjustment for sex and age we calculated an OR (95% CI) of 2.36 (1.52–3.65) for the association of haplogroup T with CAD. Further adjustment for possible confounders revealed that the strong association between haplogroup T and CAD was not explained by differences in age, sex, hypertension, body mass index, or smoking status (Table 3). Even after adjustment for multiple comparisons by Bonferroni correction the p-value remained significant. None of the other haplogroups showed a significant association with CAD (Table 3). Haplogroup frequencies were very similar in the two study centres, Salzburg and Graz. Nearly identical associations were seen when the analyses were stratified according to the two study centres from which the patients had been recruited. The age- and sex-adjusted OR (95% CI) for the association between CAD and mitochondrial haplogroup T was significant in patients recruited in Graz [n = 195; OR 2.22 (1.36–3.63); p = 0.001] and those recruited in Salzburg [n = 292; OR 2.36 (1.14–4.88); p = 0.021]. In contrast, no difference in the frequencies of mitochondrial haplogroups was seen between patients with valvular heart disease with documented absence of CAD and healthy controls (Table 3).

**Table 3:** Odds ratios (OR) and 95% confidence intervals (CI) for the association between mitochondrial haplogroup and CAD and Valvular Heart Disease, respectively.

	Haplogroup	OR* (95% CI)	p-value	aOR^† ^(95% CI)	p-value
Coronary artery disease					
	H	1 (ref)		1 (ref)	
	U	1.06 (0.69–1.61)	0.791	1.18 (0.72–1.93)	0.504
	J	0.92 (0.58–1.47)	0.738	1.09 (0.63–1.89)	0.766
	T	2.36 (1.52–3.65)	< 0.0005^‡^	2.33 (1.37–3.98)	0.002^‡^

Valvular Heart disease					
	H	1 (ref)			
	U	1.05 (0.50–2.17)	0.903		
	J	0.96 (0.44–2.14)	0.930		
	T	1.24 (0.52–2.96)	0.626		

*Adjusted for age and sex (CAD n = 382, Valvular heart disease n = 93, controls n = 1203); ^†^adjusted for age, sex, smoking, hypertension, and body mass index (CAD n = 345, controls n = 1095); ^‡^Bonferroni-corrected statistical significance: p < 0.05/3 × 2 = < 0.008.

We next compared the frequencies of the mitochondrial haplogroups between patients with diabetes with retinopathy and patients with diabetes without retinopathy. Haplogroup T was significantly more prevalent in patients with diabetic retinopathy than in patients without that condition (12.1% vs 5.1%; Tables 2, 4). There was a positive association between haplogroup T and retinopathy among patients with diabetes, which became significant when the best-fitting model was created (Table 4). After adjustment for multiple comparisons by Bonferroni correction the association was no longer significant, most likely due to the small sample size. Subgroup analysis revealed no significant differences in DNA haplogroups between patients with and without proliferative diabetic retinopathy (n = 75; Pearson chi-square 3.4, df4, p = 0.493). Also, no trend of haplogroup T with severity of retinopathy was observed as can be derived from the frequencies of haplogroup T in non-proliferative retinopathy (13.3%) and proliferative retinopathy (10.8%).

**Table 4:** Odds ratios (OR) and 95% confidence intervals (CI) for the association between mitochondrial haplogroup and retinopathy in patients with diabetes (n = 179).

Haplogroup	OR* (95% CI)	p-value	aOR^† ^(95% CI)	p-value
H	1 (ref)		1 (ref)	
U	1.54 (0.64–3.69)	0.330	1.92 (0.72–5.07)	0.190
J	2.59 (0.81–8.34)	0.110	2.04 (0.50–8.36)	0.324
T	2.97 (0.93–9.48)	0.065	3.60 (1.02–12.68)	0.046^‡^

*Adjusted for sex; ^†^adjusted for sex, history of myocardial infarction, levels of HbA1c and therapy with oral antidiabetics; ^‡^Bonferroni-corrected statistical significance p < 0.05/3 = < 0.017.

## Discussion

Our data show an association of mtDNA haplogroup T with coronary artery disease and diabetic retinopathy. Mitochondrial dysfunction has been implicated in the pathogenesis of several diseases characterised by free radical-induced damage [19-21]. Genetic association of specific mtDNA haplogroups has been demonstrated with aging and multifactorial age-related diseases like Alzheimer's disease, multiple sclerosis and cancer [22]. Although the precise mechanisms underlying the associations of mtDNA haplogroups with multifactorial age-related diseases including CAD and diabetic retinopathy remain speculative, haplogroup-specific differences in free radical production during OXPHOS may be one such mechanism. Thus, presence of haplogroup T might be associated with increased oxidative stress or increased susceptibility to oxidative stress. Individuals carrying haplogroup T might be more vulnerable to oxidative damage than carriers of other haplogroups.

While intracellular ROS serve mainly for host defence against infectious agents, redox-sensitive signal transduction, and other cellular processes, the extracellular release of ROS may damage surrounding tissues, potentially promoting inflammatory processes [23,24]. ROS are involved in aging and many diseases such as atherosclerosis, cancer, diabetes mellitus, neurological degeneration, and tumor invasion. There is strong evidence that inflammation also plays a major role in the etiology and progression of diabetic retinopathy [14]. Inflammation is an integral part not only of CAD but also of other complex diseases like multiple sclerosis, where an association of mtDNA haplogroups has been reported [25]. Enhanced formation of ROS may affect four fundamental mechanisms that contribute to atherogenesis, namely: oxidation of low density lipoprotein (LDL), endothelial dysfunction, vascular smooth muscle cell growth, and monocyte migration [21]. Interestingly, we did not find an association of haplogroup T to markers of inflammation like C-reactive protein (CRP) and LDL (data not shown).

Previous exact analysis of specific differences in the mtDNA haplogroups revealed that haplogroup T is generally associated with a number of polymorphisms in the non-coding control region (16126, 16294), mitochondrial ribosomal RNAs (709, 1888), mitochondrial transfer RNAs (10463, 15928), several SNPs not changing amino acids in the corresponding proteins, and a polymorphism at position 4917 leading to a change of asparagin to aspartate in the ND2 protein [26]. All these SNPs could contribute to altered OXPHOS performance and ROS production. This hypothesis was substantiated by Ruiz-Pesini et al., who have shown that sperm cells of individuals with haplogroup T have a lower activity of respiratory chain complexes than sperm cells with haplogroup H [5]. In contrast, another study was not able to demonstrate bioenergetic differences of isogenetic cell lines representing haplogroup H and T [27]. Unfortunately alteration of ROS production in these cell lines with different haplogroups has not been reported. We assume that the differences in ROS production among the haplogroups are subtle, requiring both time and interactions with other genetic and environmental factors before pathological manifestation. In accordance with previous studies, we found no association between mtDNA haplogroups and type 2 diabetes mellitus when all patients with diabetes (with and without retinopathy) were included [28]. Epidemiological studies in Japan showed an association of a mtDNA variation (position 5178, haplogroup N9b) with myocardial infarction [29,30]. We did not investigate the mtDNA variant located at position 5178, which is an indicator for Asian haplogroup D and the N9b haplogroup, because these variations are not prevalent in European Caucasians. Our data, however, support the concept that mtDNA variability is associated with vascular diseases.

A recent study from Denmark found no association of mtDNA haplogroups with ischemic cardiovascular disease, cancer, infectious diseases, longevity and mortality in a general population of northern European descent [31]. The study was perspective, included a large number of individuals with a long follow up. No major association to a wide variety of diseases was observed, although there is a vast literature on the association of mtDNA haplogroups with several diseases available. One explanation could be the different geographical distribution of mtDNA haplogroups. Therefore, our study on a middle European population can not be directly compared with the Danish study. Furthermore, Benn et al. investigated cases with myocardial infarction or symptoms considered characteristic of angina pectoris whereas in our study we included patients with angiographically documented CAD (i.e. ≥ 50% stenosis of at least one of the major epicadial vessels) – a criteria consistently used in the literature defining clinically relevant CAD [32,33].

We assume that a certain percentage of our control subjects will develop CAD and/or diabetes. This might have led to an underestimation of the effects we observed in the present study. If these persons could have been excluded, then one could expect that the prevalence of haplogroup T would have been even lower than was observed in the present control cohort; this in turn would increase the statistical significance of our findings. When we performed statistical analysis of CAD patients with a similar mean of age as controls, we found that there was an even larger difference in frequencies of haplogroup T between cases and controls (data not shown). We can not exclude that other unknown and unmeasured confounding factors are present.

## Conclusion

In the present study, mitochondrial haplogroup T is significantly associated with the presence of CAD and diabetic retinopathy, indicating that the presence of haplogroup T contributes to the risk of developing both CAD and diabetic retinopathy. The association between vascular disease and haplogroup variation of mtDNA, as demonstrated in the present study, should stimulate further investigation on their biological effects as well as on their relations to chronic diseases.

## Competing interests

The authors declare that they have no competing interests.

## Authors' contributions

BK, WS, RM designed the study. Patients were recruited by OS, IA, RM, MW, AH, RW, OS, BP, BI and WR. Laboratory work was undertaken by EEM, FAZ, DS, JAM and MW. Statistical analysis was done by WE and EEM. BK wrote the first draft of the paper. All other authors contributed to the final version of the manuscript and have approved the final version.

## Pre-publication history

The pre-publication history for this paper can be accessed here:


